# Red Cell Distribution Width and Mean Corpuscular Volume Alterations: Detecting Inflammation Early in Occupational Cement Dust Exposure

**DOI:** 10.7759/cureus.60951

**Published:** 2024-05-23

**Authors:** Rahnuma Ahmad, Md. Ahsanul Haq, Susmita Sinha, Halyna Lugova, Santosh Kumar, Mainul Haque, Qazi Shamima Akhter

**Affiliations:** 1 Physiology, Medical College for Women and Hospital, Dhaka, BGD; 2 Biostatistics, International Centre for Diarrhoeal Disease Research, Bangladesh (icddr,b), Dhaka, BGD; 3 Physiology, Khulna City Medical College and Hospital, Khulna, BGD; 4 Faculty of Medicine and Health Sciences, UCSI (University College Sedaya International) University Bandar Springhill Campus, Port Dickson, MYS; 5 Periodontology and Implantology, Karnavati School of Dentistry, Karnavati University, Gandhinagar, IND; 6 Research, Karnavati Scientific Research Center (KSRC) School of Dentistry, Karnavati University, Gandhinagar, IND; 7 Pharmacology and Therapeutics, National Defence University of Malaysia, Kuala Lumpur, MYS; 8 Physiology, Dhaka Medical College and Hospital, Dhaka, BGD

**Keywords:** red blood cell, prevention, blood component, early detection, oxidative stress, inflammation, toxic, heavy metals, exposure, cement dust

## Abstract

Introduction

Cement dust emitted during cement manufacture consists of toxic components. Occupational cement dust exposure may cause inflammation in the human body, which may be detected early by observing changes in blood parameters such as red blood cell distribution width (RDW) and mean corpuscular volume (MCV).

Objectives

The study aims to observe the effect of occupational cement dust exposure on RDW and MCV.

Methods

This study was performed in the Department of Physiology of Dhaka Medical College, Dhaka, Bangladesh, and a factory in Munshiganj, Bangladesh, from September 2017 to August 2018. Ninety-two participants between 20 and 50 years were included (46 subjects were occupationally exposed to cement dust, and 46 were not exposed to cement dust). A pre-designed questionnaire was used for data collection. An independent sample t-test was used to analyze basic information, such as blood pressure and BMI. The multivariate regression model was used to analyze the effect of cement dust exposure on the study group. The impact of cement dust exposure duration was analyzed using the multivariate regression model. The level of significance was p < 0.05. The statistical analysis was performed using STATA-15 (StataCorp, College Station, TX), and the graphical presentation used GraphPad Prism v8.3.2.

Results

The cement dust-exposed participants had a significantly higher value of MCV by 1.19 fi (95% CI = 0.02, 4.84; p = 0.049) and a 5.92% increase in RDW (95% CI = 5.29, 6.55; p < 0.001) than that of the control group.

Conclusion

The study reveals that exposure to cement dust causes significant changes in RDW and MCV. These changes may indicate hemolysis due to inflammation.

## Introduction

The cement industry is exhibiting increasing demand around the globe, particularly in low- and middle-income countries (LMICs), due to the urbanization and development of infrastructure in these countries. However, with development comes the burden of pollution and health hazards for those within and around the factories where cement is manufactured [1-3]. Cement production, distribution, and use may lead to the emission of various toxic substances into the environment [4,5]. Exposure to cement dust, both environmental and occupational, may harm human health, causing harm to several systems of the body, including the respiratory, integumentary, and gastrointestinal systems. There are reports of the workers in this industry, as well as those living near it, suffering from inflammation, cough, emphysema, liver disease, carcinoma, and fibrosis [6,7].

The composition of cement includes several heavy metals health in varying proportions, which are hazardous to humans, like lead (Pb), cobalt (Co), cadmium (Cd), iron (Fe), manganese (Mn), chromium (Cr), arsenic (As), and nickel (Ni) [8-10]. The concentration of these heavy metals in cement in different countries has been observed to vary [11]. A study found the concentration of Cr, Ni, Mn, and Cu to be higher in cement from the United States of America (USA) than that from Nigeria. The cement from the USA had Ni concentration twice as those in Nigeria, while Cu, Cr, and Mn levels were 10, 7, and 13.4, respectively, times more than those in Nigeria. However, Pb and Hg concentrations were higher in Nigerian cement, and the concentration of Cd was about 9-11 times higher in cement dust when compared to cement from the USA [12]. The damaging effects of the toxic components of cement depend on the concentration, exposure duration, bioaccumulation, and sensitivity of the individual [13-19].

The length of distance that the wind may carry the dust produced in the process of producing cement depends on the particle size [3,20]. The size of cement dust particulate matter (PM) varies between 0.05 and 10 μm [21]. Dust in the 10-2.5 μm range may deposit within the upper respiratory tract, and dust with a diameter less than 2.5 μm can penetrate circulation [11,22,23]. The cement dust may be absorbed through the pores of the skin and the digestive system other than the respiratory route [24,25]. The first individuals to be affected are those working within the vicinity of the cement factories, particularly the workers in the packing section, as noted in several studies [11,26,27].

The toxic heavy metals can cause several harmful effects, such as exposure to hexavalent chromium, which may cause dermatitis [28]. Cd in high concentrations harms human health as the half-life on average of Cd in the body of humans ranges from four to 19 years [12]. Exposure to toxic heavy metals for a prolonged duration may result in renal tubular dysfunction, respiratory tract, and the immune system. Several studies have noted an impact on the hematological system upon exposure to cement dust [18]. Emanuel and Alabi [18] found that a decrease in hemoglobin and red blood cell concentration and higher white blood cell count in cement factory workers suggest that cement dust exposure may bring inflammatory changes and cause anemia in those exposed [29].

The heavy metals, Pb and Cd, have been found to impede various activities performed by the erythropoietic system [30]. They also bind strongly to the proteins present within the RBCs and those on the RBC membrane [31,32]. Cadmium also interferes with iron absorption from the gastrointestinal system and accumulates within the kidneys in case of chronic exposure, interfering with erythropoietin production [30,33,34]. A study reported that despite patients suffering from severe anemia, erythropoietin levels did not increase in case of chronic intoxication with Cd [35].

The red blood cell distribution width (RDW) is performed as a routine part of a complete blood count test (CBC) and is a measure (quantitative) of the size uniformity of red blood cells in circulation [30,36]. Here, the width in RDW is about the distribution curve width. The greater the value of RDW, the greater the size variability of the erythrocytes. RDW deviation from normal may indicate a rise or fall in the destruction of erythrocytes or deficiencies of nutrients. Reticulocytes (immature RBCs) are more considerable in size than mature RBCs, and their presence would increase RDW. Reticulocyte count in circulation may increase in case of hemolysis to compensate for the excess RBC loss [37]. Red blood cells are sensitive to inflammation and are likely to hemolyze following oxidative stress [38,39]. Subclinical inflammation within the systemic circulation and other dysfunctions within the body may be indicated by the determination of RDW [40]. Thus, a "high normal" value of this parameter may suggest the presence of hemolysis, inflammation, and oxidative stress. The association has been noted between all-cause mortality, high RDW, and cardiovascular morbidity [41-48]. Mortality related to SARS-CoV-2 (severe acute respiratory syndrome coronavirus 2) infection has also been linked to higher RDW [49]. This parameter has been suggested to measure mortality and disease prognosis [50-54].

As cement dust consists of toxic components responsible for causing inflammation, oxidative stress, and anemia, as noted in previous studies [18,19,26,28,30], and the link between RDW and outcomes of cardiovascular morbidity and all-cause mortality has previously been noted [41,49,52-54], this study was performed to investigate the effect of exposure to cement dust occupationally on RDW and MCV in the workers of cement-producing factory in Bangladesh.

Objective of the study

The study aims to observe the impact of being exposed to cement dust on the CBC parameters of RDW and MCV cement factory workers.

Problem statement

Previous studies have observed changes in RDW and MCV in inflammatory conditions like cardiovascular disease and SARS-CoV-2 infection [41-48]. Toxic heavy metals like Pb and Cd exposure have also led to changes in RDW and MCV values [30]. Cement dust consists of several heavy metals, including Cd and Pb, and long-term exposure to these toxic components brings about inflammatory damage to vital organs like kidneys, as has been observed in previous studies. The management of such chronic diseases may become burdensome and often impossible for the working class who are living in poverty, particularly in LMICs [29,55]. Subclinical inflammation under cement dust exposure may be determined using RDW and MCV, which are sensitive to early inflammatory changes and are accessible and affordable [30,56-58].

## Materials and methods

Study design

This study used a mixed method, with a cross-sectional study design for the quantitative part.

Study place and period

The study was conducted from September 2017 to August 2018 at Dhaka Medical College, Dhaka, Bangladesh, and a Munshiganj-based cement production mill in the country.

Study population

The study population consisted of individuals who were occupationally exposed to dust emitted during cement production in Munshiganj (study group) and those with no history of encountering dust from cement hailing from the capital city of Dhaka (control group). The participants in the study group were selected from sections of the cement factory with the most cement dust exposure, that is, the loading, crushing, milling, and bagging sections of the cement plant as has been done in previous study [18]. The control group consisted of subjects with no history of cement dust exposure who resided in different parts of Dhaka. Their travel history in the six months before the study, occupation, address of residence, and work were noted to ensure they were not exposed to this toxic dust.

Selection criteria

This study included subjects in the age range of 20-50 years. The individuals not suffering from any known acute or chronic disease and who have been in contact with dust or cement while working for a duration equal to or higher than two years were enrolled in the study group. Subjects without any chronic or acute diseases and who have not been exposed to the dust of cement were enrolled in the control group. The threshold of two years was taken because chronic inflammation has a slow onset and may take years to develop. Also, this choice of threshold was on par with the previous study on the effects of cement dust exposure on hematological parameters [18,59].

The study excluded individuals with a known history of hematological, respiratory, liver, gall bladder, renal, or respiratory disease. Subjects with a history of allergies were also excluded. Exclusion criteria also included those suffering from any malignancies, acute infections, or history of receiving iron therapy, anticoagulants, chemotherapy, or blood transfusion within three months before the conduct of this research. The exclusion criteria applied to both the study and control groups.

Sampling technique

Non-randomized purposive sampling was performed in this study.

Sample collection

Blood was drawn (three milliliters [mL]) from the antecubital vein and added to a test tube containing anticoagulant ethylenediaminetetraacetic acid (EDTA) to observe the study participants' RDW and mean corpuscular volume (MCV). The expression of RDW is in percentage and is the coefficient of RBC size variation. To obtain the value of RDW, the volume standard division of RBC is divided by MCV. An automated hematology analyzer (Horiba Pentra DX) was used to determine these parameters. The blood samples were analyzed in the Laboratory Medicine Department of Dhaka Medical College Hospital, Dhaka, Bangladesh.

The automated hematology analyzer used in this study was the Horiba Pentra DX hematology analyzer. A study was carried out in 2010 by Hur et al. in the Department of Laboratory Medicine at the Konkuk University School of Medicine, Konkuk University Hospital, to assess the validation of the automated hematology analyzer by manual slide review. They noted that when the manual method was taken as standard, the sensitivity and specificity were 89.8% and 93.7%, respectively, in Horiba Pentra DX [60].

Another study, performed by Kim et al. in 2013 to evaluate umbilical cord blood by assessing CBC parameters with hematology analyzer ABX Pentra DX 120 and Sysmex XE-2100, found that the ABX Pentra DX hematology analyzer exhibited better correlation with manual count for mononuclear cells and showed better-flagging performance. They suggested that ABX Pentra DX may be valuable for evaluating the quality of cord blood [61].

Data collection

Data was collected using a structured questionnaire that was answered by the study population after meeting the inclusion criteria. Open-ended and closed-ended questions were included in the questionnaire. Closed-ended questions were employed to obtain quantitative data, while open-ended questions and direct conversations were used to collect qualitative data.

This study's questionnaire was adapted from the standard questionnaire of the Occupational Safety and Health Administration (OSHA) [62,63] and modified Kuppuswamy socioeconomic scale [64]. The questionnaire was modified to consider the inclusion and exclusion criteria and the country's cultural background. The questionnaire was divided into three sections. In the first section for both the study and control groups, the basic information concerning personal history (age, sex, and locality) was obtained. The history of alcohol intake, smoking history, history of drug intake, medical history, and family history, including a history of chronic diseases like diabetes mellitus, hypertension, and bronchial asthma, were also collected. Control subjects' address of residence, work, and travel history within six months before this study was noted to ensure there was no exposure to cement dust.

The following drug consumption and medical histories were collected from the participants to ensure the inclusion of subjects without chronic diseases and not undergoing any therapies that may affect the hematological system (Table 1).

**Table 1:** Depicting medication and medical (disease) history regarding the selection of this study participants

Medication history	Medical (disease history)
Antihypertensive	Cardiovascular disease
Antidiabetic	Bronchial asthma
Steroid	Diabetes mellitus
Bronchodilator	Allergy
Vitamin supplementation	Chronic obstructive pulmonary disease
Iron therapy	Thalassemia
Anticoagulant	Iron deficiency anemia
Anti-allergic	Renal disease
Chemotherapy	Liver disease
-	Malignancy
-	Acute infection
-	Undergoing surgery
-	History of blood transfusion reception within three months before study

In the second section of the questionnaire, a cement dust exposure duration history was taken (for subjects exposed to cement dust). The knowledge of the cement dust-exposed subjects regarding the usage of personal protective gear and the effects of cement dust exposure on health was also noted with the questions: "What is your understanding about the use of PPE?" and "How do you think the exposure to cement dust affects your health?"

The final section of the questionnaire included a physical examination of the participants. This included anthropometric measurements, such as height, weight, and BMI, and general and systemic physical examinations, such as pulse and blood pressure recording. A face-to-face interview was conducted to complete the questionnaire. The participants in the study group were selected from the sections of the cement factory with the most cement dust exposure, that is, the loading, crushing, milling, and bagging sections, as has been done in previous studies [18].

The control group consisted of subjects with no cement dust exposure history who resided in different parts of Dhaka. Their travel history in the six months before the study, occupation, address of residence, and work were noted to ensure they had not been exposed to this toxic dust. The participant's data have been kept anonymous using codes.

Ethical approval

Ethical approval for this study was obtained from the Research Review Committee and Ethical Review Committee of Dhaka Medical College, Dhaka-1000, Bangladesh (Reference No.: MEU-DMC/ECC/2018/06, dated 02.01.2018). The researchers explained their research goal and future publication plans to the participants. Each of the study participants was informed of the research details and procedures to be performed, and they gave written consent after receiving the information.

Impact of this research work

This study will help find a simple, easily accessible, and inexpensive test parameter to detect early inflammatory changes in the body resulting from exposure to cement's toxic dust while working in cement production. This may help take preventive measures for those exhibiting such changes at an early stage to prevent further organ damage. An easily obtainable and affordable test may aid the population working in these factories and living in poverty to avoid health damages for which they may not be able to afford treatment after long-term exposure to this toxic dust. This study will also encourage the company owners and policymakers to implement such tests as part of their routine health checkups for these factory workers.

Statistical analysis plan

**Figure 1:**
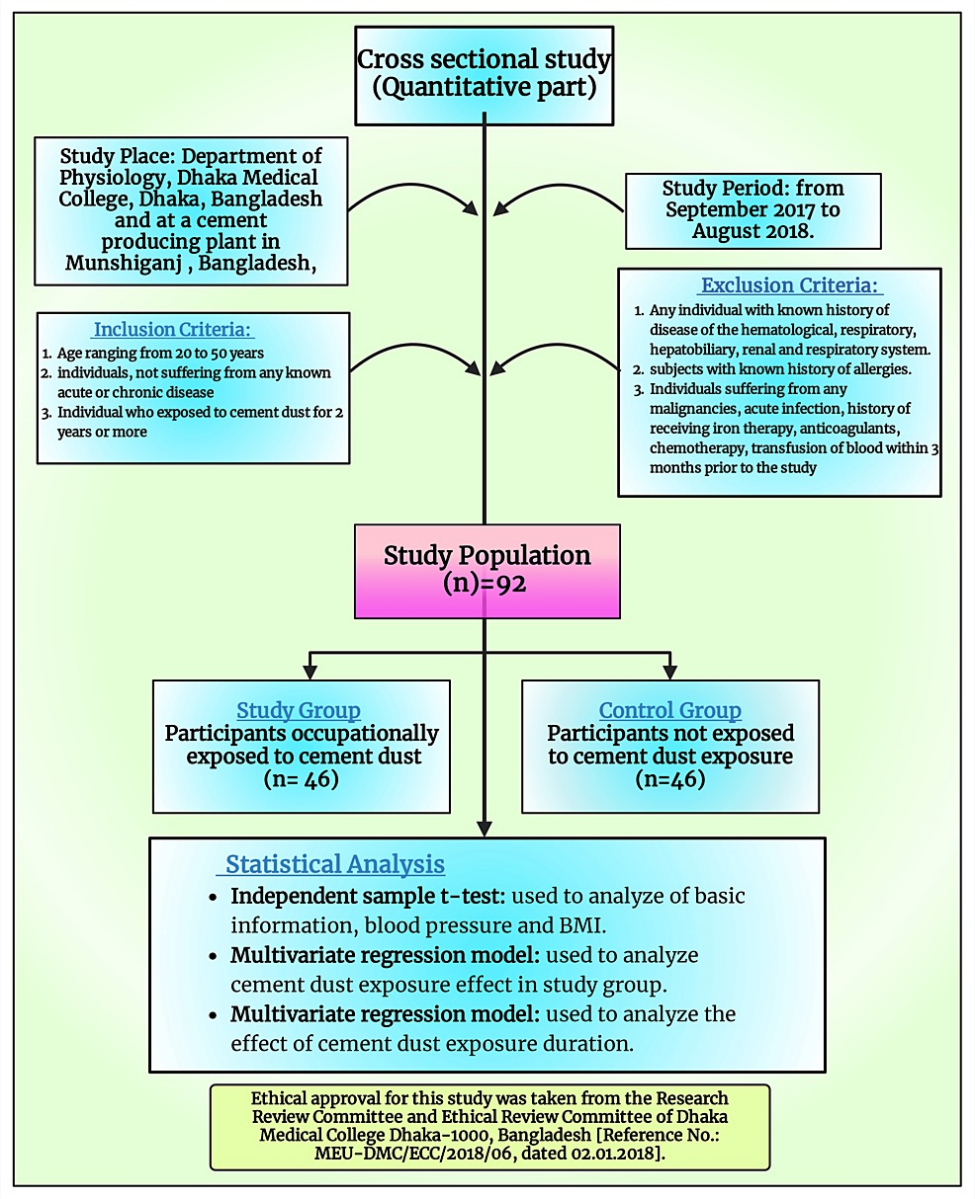
Materials and methods employed in this study Notes: This figure was drawn using the premium version of BioRender (https://biorender.com/ accessed on May 6, 2024) with license number BP26SASKLS [65]. Image credit: One of the authors, Susmita Sinha.

A descriptive analysis was done for the determination of demographic characteristics. We used an independent sample t-test for continuous variables and cross-tabulation for categorical variables (BMI) to check the distribution of essential information and blood pressure. We used a multivariate regression model to check the effect of exposure (study group) compared to control group participants. The regression model was adjusted by age and BMI (category). To further assess the effect of duration of exposure (years) among the study group participants, we also implied a multivariate regression model, which was adjusted by age and BMI. In the analysis, we considered p < 0.05 as significant. The statistical analysis was performed using STATA-15 (StataCorp, College Station, TX), and the graphical presentation used GraphPad Prism v8.3.2. The materials and method of this study are shown in Figure 1.

## Results

The baseline information of the enrolled individuals was examined and presented in Table 2, where the study group comprised 46 individuals, and an equal number constituted the control group. The average age for both groups was comparable, with the study group having a mean age of 33.2 years (±8.37) and the control group averaging 33.5 years (±7.96). The distribution of BMI categories revealed that 35 (76.1%) of the study group had a normal BMI, while 11 (23.9%) were overweight. In the control group, 29 (63.0%) had a normal BMI, and 17 (37.0%) were classified as overweight. Additionally, the blood pressure measurements showed slight variations between the groups, with the study group having higher mean systolic (117.7 ± 15.2) and diastolic (71.7 ± 9.61) blood pressure compared to the control group (113.0 ± 12.2 and 70.9 ± 9.21, respectively). The participants in the study group had an average work exposure of 7.17 years in the cement factory.

**Table 2:** Baseline information of the enrolled individuals. Notes: Data was presented as mean ± SD or number with percentage in the parenthesis. To estimate the p-value, we used an independent sample t-test for continuous observation and the chi-square for 2x2 contingency observation. SBP: Systolic blood pressure; DBP: Diastolic blood pressure.

	Case (n = 46)	Control (n = 46)	p-value
Age	33.2 ± 8.37	33.5 ± 7.96	0.839
BMI, kg/m^2^			
Normal	35 (76.1%)	29 (63.0%)	0.174
Overweight	11 (23.9%)	17 (37.0%)	
SBP, mmHg	117.7 ± 15.2	113.0 ± 12.2	0.108
DBP, mmHg	71.7 ± 9.61	70.9 ± 9.21	0.659
Duration of exposure, years	7.17 ± 2.82	-	-

**Figure 2:**
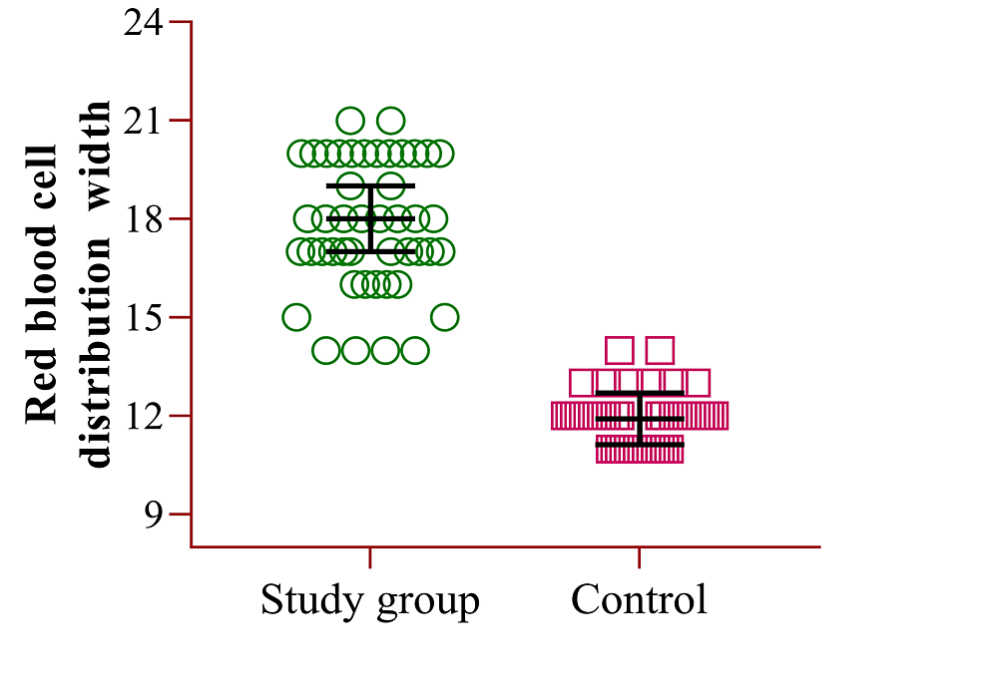
CBC parameter of red blood cell distribution width (RDW) CBC: Complete blood count.

Figure 2 shows the CBC parameter of RDW, which is reported as 17.8% (±1.99) for the study group and 11.9% (±0.78) for the control group.

**Figure 3:**
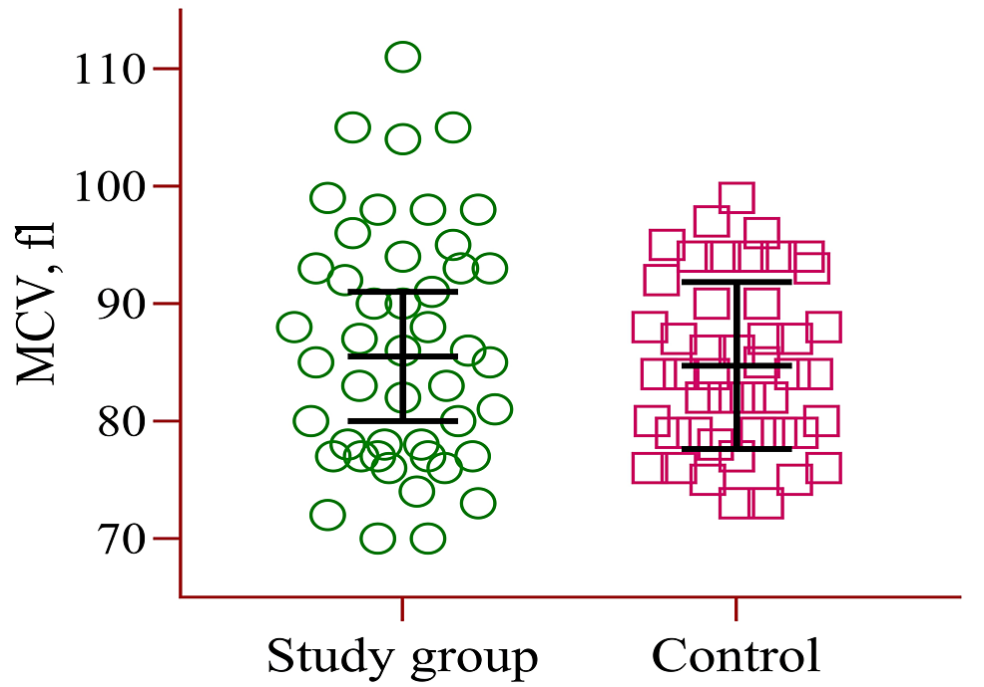
CBC parameter of mean corpuscular volume (MCV) CBC: Complete blood count.

Figure 3 shows the CBC parameter of MCV with averages of 86.3 fl (±10.2) for the study group and 84.7 fl (±7.11) for the control group.

Participants in the study group exhibited a significant increase of 1.19 femtoliters in MCV (95% CI: 0.02, 4.82; p = 0.049) and a 5.92% increase in RDW (95% CI: 5.29, 6.55; p < 0.001) in comparison to participants in the control group (Table 3).

**Table 3:** The effect of working in a cement factory on CBC parameters compared to control participants Notes: The multivariate regression model was used to estimate the p-value, and the regression model was adjusted by age and BMI (category). Data was presented as regression coefficient (β) and 95% confidence interval, and a p-value of <0.05 was considered significant. CBC: Complete blood count.

Study group	β-Coeff (95% CI)	p-value
MCV, fi	1.19 (0.02, 4.82)	0.049
RDW, %	5.92 (5.29, 6.55)	<0.001

**Figure 4:**
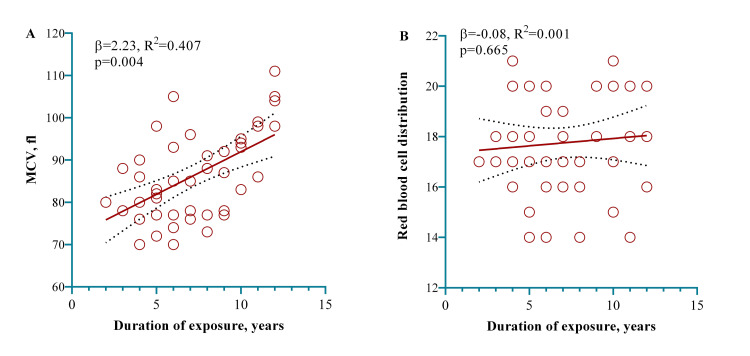
A model showing that one year of exposure working in a cement factory significantly increased the MCV Notes: We examined the linear relationship between the duration of exposure and two blood parameters, MCV (A) and RDW (B). The red line indicates the linearity of this relationship, with red dots representing individual observations. A multivariate regression model was employed to calculate the p-value, adjusting for age and BMI (categorical) in the regression model. MCV: Mean corpuscular volume; RDW: Red blood cell distribution width.

We used a multivariate regression model to check the further association with the duration of exposure within the case group participants. The model showed that one year of exposure working in a cement factory significantly increased the MCV by 2.23 fi (p = 0.004) as shown in Figure 4.

## Discussion

This study's findings indicate a link between occupational exposure to cement dust and an increase in MCV and RDW. A significant positive correlation was also noted between the duration of exposure to cement dust and MCV. We also observed that the increase in these parameters was independent of the sociodemographic characteristics of the participants. An increase in RDW and MCV indicates subclinical inflammatory conditions in the body [30,66].

Shanshal and Al-Qazaz reported that cement factory workforces exhibited statistically significant (p < 0.001) shrunken lung function and atypical readings of spirometry when compared with non-exposed (control) to cement dust [67]. Omidianidost et al. similarly reported that cement dust exposure leads to poor pulmonary function, especially affecting the peak expiratory flow (PEF) variable [68]. Rodríguez-Zamora et al. revealed in their study that in Costa Rica, workers of grain warehouses exhibited increased respirable dust molecule concentrations, exceeding the international standard cut-off point [69]. Bama et al. reported that renal physiology is significantly impaired (p < 0.05) with an acquaintance with construction rock dust among Indian cases [70].

Heavy metals in street dust represent a risk to human health due to their toxicity, persistence, and bioaccumulation. The street dust in such cities is contaminated by arsenic (As), cadmium (Cd), chromium (Cr), copper (Cu), mercury (Hg), manganese (Mn), nickel (Ni), lead (Pb), and zinc (Zn) beyond the median levels of the world soil background values [71]. The United States America (USA) Environmental Protection Agency’s health risk evaluation model showed that the total heavy metal health risk levels in the road dust ranged from 5.71 × 10^-3^ (adult) to 2.57 × 10^-2^ (children), with an average risk of 7.35 × 10^-2^ [72]. Heavy mercury (Hg) air pollution has been reported in the vicinity of compact fluorescent lamp (CFL) plants and Hg mining areas [73,74]. Another study reported that Hg acts as a proponent carcinogen [75]. Liu et al. revealed the presence of high-level barium (Ba), Pb, and Cu in non-carcinogenic health in street dust, especially in high-traffic volume regions [76]. In the welding industry, workers are exposed to substantial quantities of metal dust and fumes containing Mn, aluminum (Al), Ni, and chromium/hexavalent chromium (Cr(VI)) [77,78]. It has been reported that transient and prolonged-standing exposure to Cr dust causes respiratory difficulty and carcinoma of the pulmonary tree, respectively [79]. Rice mill personnel are commonly exposed to rice dust flecks encompassing spores, microbes, elements, and endotoxins in workstations. Thus, common workforces of Bangladesh [80] and India [81] often suffer respiratory disease from respiratory diseases and poor pulmonary physiology compared to non-exposed individuals (control).

Effects of cadmium and lead on RDW and MCV

Being exposed to toxic heavy metals like Cd and Pb, components of cement dust, may be responsible for causing inflammation and hemolysis, leading to anemia and an increase in RDW. A study performed by Peters et al. in 2019 using participants from the National Health and Nutrition Examination Survey to note the impact of Pb and Cd exposure from the environment on RDW and MCV obtained similar results. Following adjustment with sociodemographic and health factors, they also found a significant increase in RDW, as noted in our study. They suggested exposure to these heavy metals resulted in RBC breakdown due to oxidative stress, leading to an eventual increase in RDW [30].

RDW as a marker of inflammation and anemia

Horta-Baas and Romero-Figueroa suggested that RDW may be a "surrogate biomarker of inflammation" [82]. Their study measured RDW and hemoglobin in joint disease with and without inflammation and other markers of inflammation like C-reactive protein and erythrocyte sedimentation rate. They observed that patients with joint disease and inflammation had a significantly higher value for RDW and advised its use to distinguish between conditions with and without inflammation [82]. A study of hematological and biochemical parameters was performed on artisans working in welding, battery repair, selling of petrol, tin mining, and painting of cars in Nigeria in the age group of 18-60 years. These workers were exposed to heavy metals, including Pb and Cd, and reported a significant increase in liver enzymes such as alanine transaminase (ALT), aspartate transaminase (AST), and alkaline phosphatase (ALP). They also observed a substantial increase in MCV and RDW and a considerable fall in mean corpuscular hemoglobin concentration (MCHC) and RBC, indicating anemia. They suggested that oxidative stress from occupational exposure to heavy metals was both hepatotoxic and hematotoxic. A fall in RBC in the study subjects was attributed to hemoglobin auto-oxidation induced by oxidative stress, which may have led to the peroxidation and hemolysis of the RBC membrane. The increase in RDW among the artisans in their study indicated macrocytic anemia. A surge in MCV may have been influenced by vitamin B12 and/or folate deficiency. In our research, the increase in MCV may have been due to a combined impact of vitamin B12 deficiency and the effect of toxic cement dust exposure [83]. WASH (water, sanitation, and hygiene) status and ongoing regular anthelminthic with vitamin A program throughout the country improve the overall nutritional status, and anemia decreased among school-going children and adolescents [84,85].

The elevation of RDW due to exposure to cement dust in our research work may suggest its impact on the health of those exposed as there is an increasing number of research works implicating this parameter as a mortality marker [50-53]. A study noted a 23% increase in mortality risk with a percentage increase in RDW [86]. These cement dust-exposed subjects are also prone to developing anemia as a surge in RDW is a characteristic of anemia. Anemia was reported in subjects with industrial exposure to cadmium in a study conducted in Japan [30]. Anemia may result from impairment of erythropoietin formation as heavy metals like cadmium deposit in the kidney and liver and hamper erythropoietin production [87,88].

Heavy metals in cement dust link to hemolysis, oxidative stress, reactive species production, and inflammation

Another component of cement dust, lead, may decrease erythrocyte lifespan and inhibit heme synthesis [89,90]. Lead inhibits ferrochelatase (by acting on bone marrow erythroblast) and delta-aminolevulinic acid of RBCs, leading to inhibition of the formation of heme [91-93]. This heavy metal also affects RBC maturation by altering erythropoietin formation [94,95]. Lead promotes hemolysis by causing a deficiency of pyrimidine 5'-nucleotidase [96,97]. Ukaejiofo et al. reported significantly lower levels of hemoglobin (p < 0.0001) and packed cell volume (p < 0.05), with a significantly higher reticulocyte percentage (p < 0.05) in occupational lead handlers compared to the lead unexposed control group. They mentioned that the probable cause for the significant increase in reticulocyte percentage was the bone marrow compensating for the decrease in red blood cell mass [98].

Another study performed by Kargar-Shouroki et al. on lead-exposed Iranian battery workers observed a significant decrease in hemoglobin, RBC, PCV, MCV, mean corpuscular hemoglobin, and mean corpuscular hemoglobin concentration in lead-exposed subjects when compared to the unexposed control subjects. They concluded that exposure could disturb the hematological system of the body [99]. A research work done by Ogbenna et al. in Nigeria on 66 men occupationally exposed to lead, who repaired batteries, with an average lead exposure duration of 23.33 ± 11.03 years found a significant negative correlation of RBC count (r = −0.322; p = 0.008) and positive correlation between MCV (r = 0.277; p = 0.025) and blood lead levels. Subjects with blood lead levels more than 40 µg/l had higher values of MCV (p = 0.038). The researchers mentioned that reticulocyte count was not performed in their study, which is the study's limitation. They suggested that a change in reticulocyte count may explain the increase in the MCV value [100]. Ahmad et al. reported a significant presence of anemia among subjects with higher mean blood lead levels than those without anemia. This study was carried out in Bangladesh on lead acid battery workers, and it was noted that out of the 118 blood samples, 28% were anemic, of which 21.1% suffered from macrocytic anemia [101]. Rodents also exhibited hemolysis induction upon cadmium exposure [34]. In combination, exposure to lead and cadmium may increase lead ion bioavailability in heme pathway enzymes and, therefore, have a multiplicative effect [30,91].

Heavy metals in the cement dust could form highly reactive species like free radicals and cause DNA damage, protein depletion, sulfhydryl group depletion of proteins, and lipid peroxidation. These metals promote the disruption of cell redox systems by generating reactive nitrogen and oxygen species. The exogenous metals displace endogenous metals from protein ligands from carrier proteins. They also aggravate hydroxyl ions production through the Fenton reaction with the eventual generation of nitrite [66]. Copper, chromium, cadmium, and nickel exhibit active participation in lipid peroxidation with free radicals’ formation, which is linked to pathologies like ischemia, atherosclerosis, neurotoxicity, and nephrotoxicity [66,102-106]. Metal complexes of copper (II), manganese (II), and zinc (II) have the property of promoting redox reactions and are, therefore, the most active and cause cytotoxicity [107].

**Figure 5:**
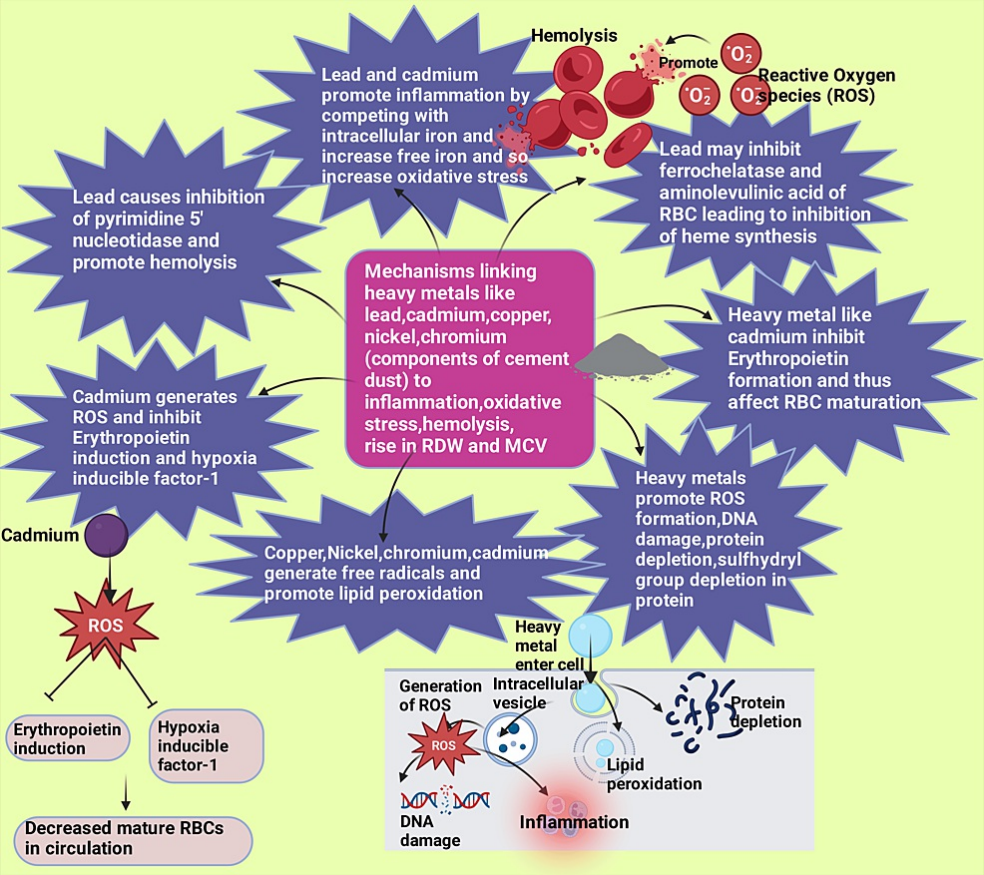
Pathophysiology resulting from exposure to heavy metals in the cement dust Notes: Heavy metals cause the formation of reactive oxygen species, DNA damage, protein depletion, lipid peroxidation, and inflammation. These metals may also inhibit heme synthesis, RBC maturation, and oxidative stress-induced hemolysis. This figure was drawn with the premium version of BioRender (https://biorender.com/ accessed on May 6, 2024) with license number WV26SANIUI [65]. Image credit: One of the authors, Rahnuma Ahmad. RBC: Red blood cells; DNA: Deoxyribonucleic acid.

Lead and cadmium also promote inflammation by competing with intracellular iron, increasing the quantity of free iron, which is unbound and can cause acceleration of intermediates of reactive oxygen species employing H_2_O_2_ (hydrogen peroxide)/metal/superoxide system [35,108,109]. Cadmium has been related to the disruption of an antioxidant system [110]. Erythropoietin induction and hypoxia-inducible factor-1 (a factor that plays a substantial part in ischemic cardiovascular disease prevention) have been noted to be inhibited by cadmium through reactive oxygen intermediate generation [35,88,111]. These reactive oxygen species may cause oxidative hemolysis [112]. Lead increases superoxide dismutase and glutathione peroxidase activity as the enzymes maintain the balance of oxidative-reductivity (Figure 5) [113].

The link between inflammation and RDW

Production of reactive oxygen species above antioxidants within the body leads to oxidative stress, which plays a role in all steps of inflammation: release of endogenous signal molecules from damaged tissue, sensing of these molecules by toll-like receptors and NOD-like receptors, and initiation of adaptive immune response through activation of signaling pathways [114]. Lippi et al. observed an independent, graded, and significant association between C-reactive protein (a marker of inflammation) and RDW [115]. RDW was also noted to have an association with CRP level and mortality in coronary artery disease patients by Lappe et al. [116]. Förhécz et al. reported that RDW values exhibited an association with inflammation and mortality in chronic heart failure patients [117]. Another study by Sembe et al. to find if inflammation and antioxidants could predict the values of RDW reported higher levels of interleukin-6 in individuals with higher RDW values [118]. Inflammation-induced hemolysis following oxidative stress would result in a compensatory increase in reticulocyte count in circulation, which would be reflected by the increase in the RDW value. Although there was a positive association between RDW and duration of cement dust exposure, it was not significant. This may have been due to the study being carried out on a sample population of a single factory with an exposure duration of 2-12 years. A study with a longer duration of exposure on a larger population involving multiple cement factories may produce a more significant result. A survey by Rajanayagam et al. noted a significant positive correlation between RDW and occupational quarry dust exposure. They included several stone quarries and more study subjects (n = 75) [119]. A significant increase in RDW in the presence of inflammation-causing agents has been noted in other studies, such as the outcome observed in our study [30,37,38,83,100,119].

Rajanayagam et al. noted a significant increase in RDW and MCV in quarry dust-exposed subjects and a significant positive association between MCV and duration of dust exposure. The positive correlation between RDW and occupational quarry dust exposure is similar to our study. However, unlike our study, they observed a significant positive correlation between RDW and duration of dust exposure [119]. This difference may be due to the number of subjects and the number of factories being more in their study, while our research was carried out in a single factory with a smaller study population.

In Nigeria, Ogbenna et al. performed a study on occupationally lead-exposed subjects with an average duration of lead exposure of 23.33 ± 11.03 years. They noted a positive correlation between MCV (r = 0.277; p = 0.025) and blood lead levels. The more the exposure, the higher the MCV value [100]. Although our study could not assess the blood levels of cement dust to correlate between MCV and blood cement toxic component levels, our study noted a significant increase in MCV with increasing duration of toxic dust exposure, which may indicate increasing levels of harmful chemicals in blood having a deteriorating effect in the hematological system. Bot et al. studied the hematological parameters of artisans working in welding, battery repair, petrol selling, tin mining, and car painting in Nigeria and observed a significant increase in MCV and RDW. These findings were similar to those of our study, suggesting that the RDW surge may indicate macrocytic anemia [83]. The study performed by Peters et al. observed an increase in RDW upon occupational exposure to cadmium and lead. They noted a positive correlation between blood cadmium concentration and an increase in RDW. Similarly, blood lead levels also showed a positive correlation with RDW levels. This study also noted higher RDW in cement dust-exposed subjects (containing cadmium and lead, among other heavy metals) than in the control. Our study did not assess individual heavy metal levels due to financial and technical constraints. Therefore, a correlation between individual heavy metals and RDW could not be performed [30].

**Figure 6:**
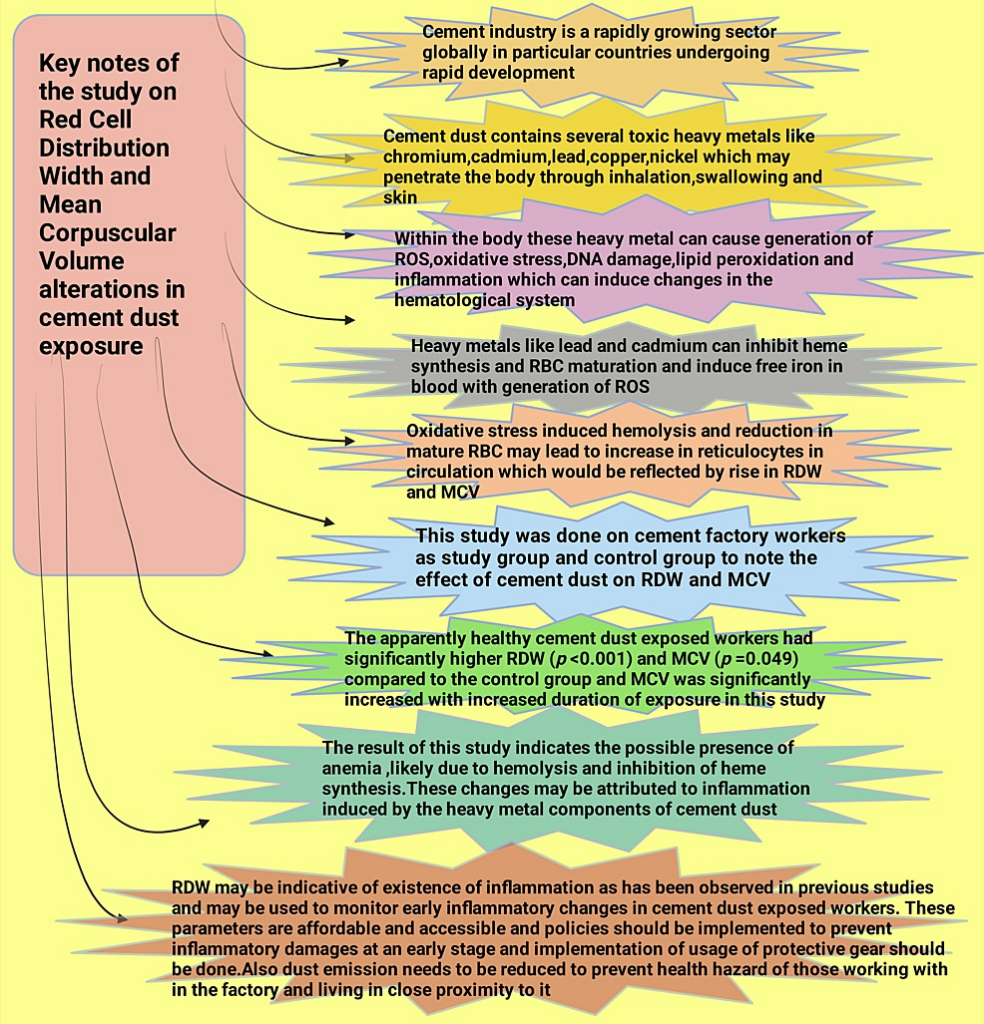
Principal findings of the study Notes: This figure was drawn using the premium version of BioRender (https://biorender.com/ accessed on May 6, 2024) with license number EZ26SEKZEH [65]. Image credit: One of the authors, Rahnuma Ahmad. RBC: Red blood cells; DNA: Deoxyribonucleic acid; ROS: Reactive oxygen species; RDW: Red blood cell distribution width; MCV: Mean corpuscular volume.

A reticulocyte count can be used as a clinical indicator for anemia. An increase in reticulocytes may occur in several anemias, including hemolytic anemia [37]. An increase in reticulocyte count may be attributed to bone marrow attempting to compensate for decreased red blood cell mass [98]. Ogbenna et al. found an increase in MCV on occupational exposure to heavy metals, but their study did not perform a reticulocyte count. This was a limitation of their research, like this study, and they suggested that an alteration in reticulocytes may have caused the increase in MCV [100]. Mohanty et al. measured the RBC oxidative stress using heme degradation product (HDP) levels. They observed a negative association (p < 0.0001) between HDP and RBC deformability. Reduction in RBC deformability causes RBC removal from circulation. They suggested that oxidative stress also promotes RBC uptake by macrophages and removes RBC from circulation [38]. Therefore, oxidative stress may have contributed to a decrease in RBC in circulation followed by anisocytosis and an increase in RDW, indicating the hematopoietic system attempting to compensate for the reduction of RBC in circulation in our study. Key findings of the study are summarized in Figure 6.

Limitations of the study

This study has certain limitations. As this research is cross-sectional, we could not determine the cause-effect pathway. As the changes in RBC occur at a subcellular level, the precise time when the physical alteration occurs in the blood cell structure could not be observed. The relationship between individual components of cement dust with RDW was not performed due to financial and technical limitations. Due to time and financial constraints, the time- and dose-dependent nature of the mechanism of action for cement dust has not been carried out.

Recommendations for future research

This study was done in one factory in the country that manufactures cement. A broad-scale, long-term research needs to be carried out in the country and globally to understand the pathways linking cement dust exposure and RDW. The connection between the individual cement dust components, RDW, and MCV may also be sought. Policymakers and owners of cement manufacturing plants should know the significance of parameters like RDW to determine early inflammatory changes in individuals exposed to cement dust. They should be encouraged to include the performance of CBC, which provides for RDW for the workers in their factories, and assign workers to parts of the plant in which there is less exposure to dust in case changes are noted in their values of RDW. Policymakers and factory owners must also use protective gear like gloves, masks, and helmets to ensure minimum exposure to toxic dust [120]. Dust emissions from these factories should also be monitored, and steps should be taken to reduce this emission [121].

## Conclusions

Cement contains several heavy metals that may cause diseases within the body by causing disharmony in body homeostasis through oxidative stress and inflammation. Observing CBC parameters like RDW and MCV may reflect early inflammatory changes within the body. RDW increases in case of subclinical inflammation and oxidative stress-induced hemolysis. As various components of cement aggravate inflammation and other pathophysiology that induce hemolysis, it is likely to increase RDW values. Our study findings indicate that cement dust exposure may cause alterations in RDW and MCV. Early detection of inflammation can aid in early diagnosis management and help to take preventive measures to safeguard the lives of these occupational cement dust-exposed subjects. RDW is a blood parameter that is affordable, accessible, and a potential marker for inflammation. It can be easily observed regularly by the owners of these cement plants and is easy for the authorities to implement. As prolonged exposure would lead to organ damage, making its management complex and expensive, it is better to monitor RDW and MCV and consider preventive measures and early management accordingly.
